# Real-world utilization of guideline-directed genetic testing in inherited cardiovascular diseases

**DOI:** 10.3389/fcvm.2023.1272433

**Published:** 2023-10-17

**Authors:** Mauro Longoni, Kanchan Bhasin, Andrew Ward, Donghyun Lee, McKenna Nisson, Sucheta Bhatt, Fatima Rodriguez, Rajesh Dash

**Affiliations:** ^1^ Global Medical Affairs Organization, Illumina, Inc., San Diego, CA, United States; ^2^ HealthPals Inc., Redwood, CA, United States; ^3^ Division of Cardiovascular Medicine and Cardiovascular Institute, Stanford University, Stanford, CA, United States

**Keywords:** real-world evidence, practice guidelines, genetic testing, cardiovascular disease, cardiomyopathy, long QT syndrome, hereditary amyloidosis, familial hypercholesterolemia

## Abstract

**Background:**

Cardiovascular disease continues to be the leading cause of death globally. Clinical practice guidelines aimed at improving disease management and positively impacting major cardiac adverse events recommend genetic testing for inherited cardiovascular conditions such as dilated cardiomyopathy (DCM), hypertrophic cardiomyopathy (HCM), long QT syndrome (LQTS), hereditary amyloidosis, and familial hypercholesterolemia (FH); however, little is known about how consistently practitioners order genetic testing for these conditions in routine clinical practice. This study aimed to assess the adoption of guideline-directed genetic testing for patients diagnosed with DCM, HCM, LQTS, hereditary amyloidosis, or FH.

**Methods:**

This retrospective cohort study captured real-world evidence of genetic testing from ICD-9-CM and ICD-10-CM codes, procedure codes, and structured text fields of de-identified patient records in the Veradigm Health Insights Ambulatory EHR Research Database linked with insurance claims data. Data analysis was conducted using an automated electronic health record analysis engine. Patient records in the Veradigm database were sourced from more than 250,000 clinicians serving over 170 million patients in outpatient primary care and specialty practice settings in the United States and linked insurance claims data from public and private insurance providers. The primary outcome measure was evidence of genetic testing within six months of condition diagnosis.

**Results:**

Between January 1, 2017, and December 31, 2021, 224,641 patients were newly diagnosed with DCM, HCM, LQTS, hereditary amyloidosis, or FH and included in this study. Substantial genetic testing care gaps were identified. Only a small percentage of patients newly diagnosed with DCM (827/101,919; 0.8%), HCM (253/15,507; 1.6%), LQTS (650/56,539; 1.2%), hereditary amyloidosis (62/1,026; 6.0%), or FH (718/49,650; 1.5%) received genetic testing.

**Conclusions:**

Genetic testing is underutilized across multiple inherited cardiovascular conditions. This real-world data analysis provides insights into the delivery of genomic healthcare in the United States and suggests genetic testing guidelines are rarely followed in practice.

## Introduction

Cardiovascular disease (CVD) is the leading cause of death worldwide, accounting for 32% of deaths in 2019 (1). The etiology of CVD is complex and includes behavioral, environmental, and genetic risk factors (1, 2). Collectively, inherited cardiovascular conditions are relatively common and encompass a range of phenotypes such as cardiomyopathies, arrhythmias, dyslipidemias, aortopathies, and some clinical presentations of transthyretin amyloidosis; heritable cardiovascular phenotypes also occur within a constellation of vascular, metabolic and neuromuscular disorders (3–7).

For example, cardiomyopathies may have a genetic or acquired etiology, or a combination of both. Inherited cardiomyopathies include hypertrophic cardiomyopathy (HCM) and dilated cardiomyopathy (DCM), among other less common phenotypes. Estimates of genetic and non-genetic etiologies vary by study, depending on the composition of the cohort (8). Genetic cardiomyopathies are generally inherited as autosomal dominant conditions, occasionally as autosomal recessive or X-linked conditions, and are characterized by incomplete penetrance and variable expressivity. De novo pathogenic variants are less common. Furthermore, inherited CVD are associated with notable locus and allele heterogeneity (8, 9).

Genetic testing for heritable genomic variations supports many clinical decision-making activities including diagnostic confirmation, risk stratification, refinement of prognosis, individualized patient management, selection of targeted therapeutics, identification of asymptomatic or pre-symptomatic at-risk relatives, and reproductive planning. Prior studies have shown that genetic testing has a critical role in the diagnosis and management of patients with inherited cardiovascular disorders and their family members (10–18). For example, genetic testing of patients at high risk for familial hypercholesterolemia (FH) leads to improved diagnosis, initiation or modification of medical management, and improved cholesterol levels (13, 19–24). As shown by Stafford et al., genetic results changed or clarified the clinical diagnosis in 51/403 (13%) patients with pathogenic or likely pathogenic variants referred to a cardiogenetic clinic. In a subcohort of more complex cases, 26/46 (57%) received a change in management and risk stratification (25). Pediatric DCM cases with a pathogenic or likely pathogenic variant had 2.8-times increased risk of death or heart transplant, suggesting the importance of genetic testing for predicting clinical outcomes (26). In 2021, Murdock, et al. reported on a next-generation sequencing (NGS)-based genetic testing panel that returned results with clinical management implications for 32% of patients seen in institutionally affiliated adult cardiology clinics (15). In fact, the actionability of genetic information in patients with heritable cardiac conditions prompted the American College of Medical Genetics and Genomics (ACMG) to include more than three dozen genes associated with cardiovascular phenotypes on its list of secondary findings recommended to be reported (27).

Since the publication in 2011 of the first guideline recommending genetic testing for cardiomyopathies and channelopathies (28), cardiology and genetics professional practice societies around the world have increasingly recommended genetic testing for a growing number of inherited cardiovascular conditions (16, 17, 21, 24, 28–38). These clinical practice guidelines and scientific consensus statements aim to improve the diagnosis and management of patients, minimize major adverse cardiac events, and identify at-risk relatives. DCM, HCM, long QT syndrome (LQTS), hereditary amyloidosis, and FH are just a few of the conditions for which guidelines and statements recommend genetic testing for affected patients and, when a causative variant is found, their at-risk relatives. Recent progress in the pursuit of targeted therapeutics for cardiovascular conditions amplifies the importance of guideline-directed genetic testing in cardiovascular care as future clinical management of patients will increasingly rely on precise knowledge of the genetic variant(s) underlying each individual's diagnosis (33, 36, 39, 40).

Although guidelines and statements offer a rationale and evidence to support the utilization of genetic testing for patients suspected of having an inherited cardiac condition, the adoption of guideline-based recommendations in real-world clinical practice has not been thoroughly assessed (41, 42). Electronic health record (EHR) and insurance claim databases include broad, real-world, observational and longitudinal data related to patient demographics; incidence, prevalence, and natural history of disorders; comorbidities; current standards around clinical practice; treatments; outcomes; and healthcare resource utilization, including the utilization of genetic testing (41, 43, 44). Understanding how and where guideline-directed genetic testing is being adopted into routine patient care can provide actionable insights by capturing the clinical state of patients not only at the time of testing and diagnosis, but throughout their continuum of care, which can allow for prospective assessment of clinical outcomes. Real-world evidence (RWE) of genetic testing can also identify patient groups, practice settings, and geographic locations with gaps in care and unmet medical needs.

The objective of this retrospective cohort study was to understand the current landscape of guideline-directed genetic testing for select inherited cardiovascular conditions by analyzing the EHR and claims data of a large patient population with incident disease.

## Materials and methods

### Study design and data sources

The Veradigm Health Insights Ambulatory EHR Research Database linked with insurance claims data (Veradigm database) was used to perform a retrospective cohort study. The Veradigm database consists of de-identified patient records from more than 250,000 clinicians serving over 170 million patients in outpatient primary care and specialty practice settings across the United States, including internal and family medicine practitioners as well as cardiologists and other related specialties. The linked insurance claims data are sourced from United States public and private insurance providers and include enrollment data from medical and pharmacy claims.

### Study cohort

The primary cohort for this study was identified from within the Veradigm database using the inclusion and exclusion criteria specified below. Five subcohorts of inherited cardiovascular conditions were defined from within the primary cohort for patients diagnosed with DCM, HCM, LQTS, hereditary amyloidosis, or FH.

Other conditions for which clinical genetic testing is available were considered during a feasibility assessment. Brugada syndrome, arrhythmogenic right ventricular cardiomyopathy (ARVC), and catecholaminergic polymorphic ventricular tachycardia (CPVT) presented fewer than 100 cases per diagnosis in the primary cohort and were not included in the study.

The main inclusion criterion used to filter records for this study from the Veradigm database was new diagnosis of a subcohort-specific condition between January 1, 2017 and December 31, 2021, including records collected during the COVID-19 pandemic. Diagnoses were based on International Classification of Diseases, Clinical Modification, 9th (ICD-9-CM) and 10th (ICD-10-CM) revisions codes and/or structured EHR text fields (Supplementary Table 1). New diagnoses were distinguished from pre-existing diagnoses by first defining a wash-in time, which for each patient consisted of the 365 days after their first data record in the EHR or 365 days after their first enrollment date in claims, whichever occurred later.

Patients under 18 years of age were excluded. To avoid overestimating the proportion of genetic testing associated with each subcohort, additional filtering was done to exclude patients with a diagnosis of cancer or cystic fibrosis, as well as instances where a new diagnosis coexisted with a pre-existing diagnosis of another subcohort-specific condition. Patients with concurrent pregnancy were also excluded as a proxy of possible prenatal genetic testing. The codes and filters used to identify the study cohort and subcohorts are detailed in Supplementary Table 1.

### Evidence of genetic testing

Evidence of genetic testing was captured from ICD-9-CM and ICD-10-CM codes, procedure codes, and structured text fields within the Veradigm database. To most accurately capture only genetic testing intended for the subcohort-specific cardiovascular diagnosis, genetic testing associated with non-cardiac procedure codes was explicitly excluded. A one-year window (plus/minus six months) around the index date, i.e., the date when the diagnosis was recorded, was used to infer the indications of genetic testing when not explicitly stated. The use of CPT codes allowed the capture of genetic tests included on an insurance claim in either an inpatient or outpatient setting.

### Guideline codification

Genetic testing guidelines were codified for this analysis using a compilation of clinical practice guidelines and expert scientific statements (17, 24, 29, 30, 32–34). DCM, HCM, LQTS, and hereditary amyloidosis all have a class 1 recommendation (45, 46) for genetic testing; FH has a combination of recommendation classes ranging from 1-2b (Supplementary Table 2, Supplementary Table 3), indicating a strong recommendation, for which benefits are believed to significantly outweigh risks. Patients diagnosed with recognized forms of cardiomyopathy were deemed eligible of genetic testing. Because the de-identified dataset used did not include family relationships, adherence to the specified guidelines that pertain to family history were estimated by searching for genetic testing in individuals with documented evidence of a family history of the condition of interest.

### Statistical analyses

*P* values for bivariate analyses were calculated using t-tests or chi-square tests, as appropriate. *P* values were corrected for multiple testing using the two-stage step-up method of Benjamini, Krieger and Yekutieli (47). Data were analyzed using the CLINT Clinical Intelligence software analytics platform (48, 49) and Python 3.8.

## Results

**Figure 1:**
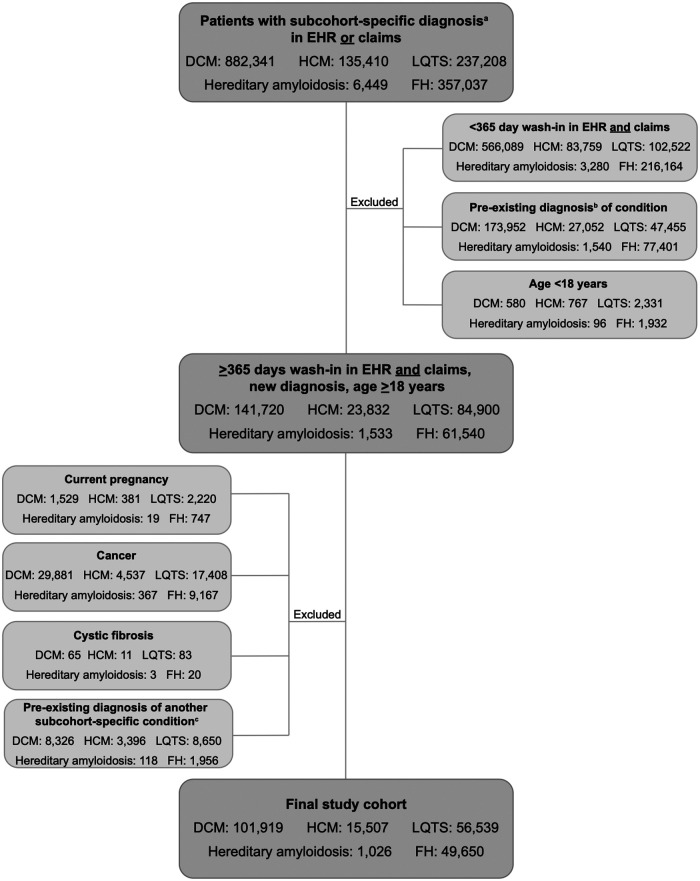
CONSORT diagram. Selection of study cohort. ^a^Diagnosis of DCM, HCM, LQTS, hereditary amyloidosis, or FH. ^b^Diagnosis of DCM, HCM, LQTS, hereditary amyloidosis, or FH within the wash-in period; ^c^Existing diagnosis of a different subcohort-specific condition. EHR, electronic health record; DCM, dilated cardiomyopathy; HCM, hypertrophic cardiomyopathy; LQTS, long QT syndrome; FH, familial hypercholesterolemia.

Of 1,618,445 patients identified in the Veradigm database with a diagnosis of DCM, HCM, LQTS, hereditary amyloidosis, or FH, 1,393,804 were excluded from further analysis for failing to meet the study criteria (Figure 1). The final study group consisted of 224,641 individuals: 101,919 with a diagnosis of DCM; 15,507 with a diagnosis of HCM; 56,539 with LQTS; 1,026 with hereditary amyloidosis; and 49,650 with FH.

Each of the five select cardiovascular diagnoses was analyzed as an independent subcohort. As shown in Table 1, only a small percentage of patients newly diagnosed with DCM (827/101,919; 0.8%), HCM (253/15,507; 1.6%), LQTS (650/56,539; 1.2%), hereditary amyloidosis (62/1,026; 6.0%), or FH (718/49,650; 1.5%) received genetic testing. At 0.8%, the DCM cohort demonstrated the lowest utilization of genetic testing. At 6.0%, the proportion of patients diagnosed with hereditary amyloidosis who received genetic testing was notably higher than other subcohorts. Neither the class of recommendation nor levels of evidence appeared to influence the percentage of patients tested across the five subcohorts.

**Table 1:** Utilization of genetic testing in patients with DCM, HCM, LQTS, hereditary amyloidosis, or FH.

Inherited cardiovascular condition	COR for genetic testing (levels of evidence)^a^	Total *N* with new diagnosis in study period	*N* (%) who received genetic testing
DCM	1 (A, B, C)	101,919	827 (0.8%)
HCM	1–2a (B-NR); n/a (A)	15,507	253 (1.6%)
LQTS	1 (B-NR)	56,539	650 (1.2%)
Hereditary amyloidosis	1 (B-NR)	1,026	62 (6.0%)
FH	1–2b (B-R, B-NR, C-EO)	49,650	718 (1.5%)
Total	** **	224,641	2,510 (1.1%)

COR, class of recommendation; n/a, not classified according to numeric COR; level A, strong evidence; level B, moderate evidence; level C, limited evidence/expert opinion; B-R, level B randomized; B-NR, level B non-randomized; C-EO, level C expert opinion; DCM, dilated cardiomyopathy; HCM, hypertrophic cardiomyopathy; LQTS, long QT syndrome; FH, familial hypercholesterolemia.

^a^ For conditions with multiple classes of recommendation or levels of evidence, additional details are provided in Supplementary Table 2 and Supplementary Table 3.

Demographics and baseline comorbidities at the time of diagnosis (index date) were analyzed to compare individuals who received genetic testing with those who did not. Patients who received genetic testing were significantly younger (*P *< 0.001 for all subcohorts) (Table 2). However, the age distribution in both groups largely overlapped, suggesting that age alone was not the primary driver of genetic testing.

**Table 2:** Patient demographics by subcohort diagnosis and evidence of genetic testing.

Total N of subcohort	DCM			HCM			LQTS			Hereditary Amyloidosis			FH		
	101,919			15,507			56,539			1,026			49,650		
Genetic testing (+/-), *P* value	+	–	*P*	+	–	*P*	+	–	*P*	+	–	*P*	+	–	*P*
*N*	827	101,092	n/a	253	15,254	n/a	650	55,889	n/a	62	964	n/a	718	48,932	n/a
Age, median (IQR)	60 (50–68)	63 (55–72)	**<0**.**001**	55 (46–64)	61 (51–70)	**<0**.**001**	51.5 (39–60)	56 (45–65)	**<0**.**001**	50.5 (39–60.8)	59 (48–69)	**<0**.**001**	55 (43–62)	58 (49–65)	**<0**.**001**
Sex assigned at birth (%)^a^															
Male	460 (56)	63,308 (63)	**<0**.**001**	127 (50)	7,288 (48)	0.66	196 (30)	21,608 (39)	**<0**.**001**	26 (42)	395 (41)	0.90	254 (35)	20,101 (41)	**0**.**005**
Female	366 (44)	37,667 (37)		126 (50)	7,950 (52)		454 (70)	34,244 (61)		36 (58)	566 (59)		464 (65)	28,776 (59)	
Not indicated	1 (0.12)	117 (0.12)	n/a	0	16 (0.1)	n/a	0	37 (0.066)	n/a	0	3 (0.31)	n/a	0	55 (0.11)	n/a
Baseline comorbidities (%)															
Coronary artery disease	598 (72)	82,588 (82)	**<0**.**001**	98 (39)	6,953 (46)	0.04	243 (37)	23,255 (42)	0.03	11 (18)	272 (28)	0.10	190 (26)	11,198 (23)	0.03
Hyperlipidemia	651 (79)	85,846 (85)	**<0**.**001**	175 (69)	11,849 (78)	**0**.**002**	388 (60)	36,785 (66)	**0**.**001**	29 (47)	717 (74)	**<0**.**001**	718 (100)	48,932 (100)	>.99
Diabetes mellitus	426 (52)	57,637 (57)	**0**.**002**	95 (38)	7,053 (46)	0.007	252 (39)	25,661 (46)	**<0**.**001**	12 (19)	514 (53)	**<0**.**001**	242 (34)	17,973 (37)	0.10
Hypertension	730 (88)	95,163 (94)	**<0**.**001**	199 (79)	13,376 (88)	**<0**.**001**	487 (75)	46,444 (83)	**<0**.**001**	34 (55)	748 (78)	**<0**.**001**	469 (65)	34,226 (70)	**0**.**008**
Family history (%)															
CVD	266 (32)	21,827 (22)	**<0**.**001**	95 (38)	2,914 (19)	**<0**.**001**	161 (25)	11,169 (20)	**0**.**003**	11 (18)	118 (12)	0.29	198 (28)	6,432 (13)	**<0**.**001**
Cardiac arrest	5 (0.6)	225 (0.22)	0.05	9 (3.6)	67 (0.44)	**<0**.**001**	13 (2)	90 (0.16)	**<0**.**001**	0 (0)	1 (0.1)	>.99	4 (0.56)	104 (0.21)	0.12
Cardiovascular symptoms (%)															
Cardiac murmur	80 (9.7)	7,050 (7)	**0**.**003**	61 (24)	2,937 (19)	0.06	64 (9.8)	4,127 (7.4)	0.02	1 (1.6)	74 (7.7)	0.13	43 (6)	2,565 (5.2)	0.42
Heartbeat abnormality	405 (49)	43,520 (43)	**<0**.**001**	122 (48)	6,788 (44)	0.26	383 (59)	31,061 (56)	0.10	24 (39)	351 (36)	0.82	209 (29)	12,019 (25)	**0**.**006**
Chest pain	578 (70)	66,446 (66)	**0**.**01**	155 (61)	8,920 (58)	0.41	425 (65)	37,749 (68)	0.26	31 (50)	476 (49)	>.99	324 (45)	18,949 (39)	**<0**.**001**
Shortness of breath/dyspnea	627 (76)	72,100 (71)	**<0**.**001**	169 (67)	9,342 (61)	0.083	418 (64)	37,581 (67)	0.12	29 (47)	506 (52)	0.46	342 (48)	17,828 (36)	**<0**.**001**
Syncope/collapse	176 (21)	16,845 (17)	**<0**.**001**	42 (17)	2,724 (18)	0.66	185 (28)	13,525 (24)	0.01	8 (13)	159 (16)	0.57	79 (11)	3,929 (8)	**0**.**005**
Abnormal blood pressure	116 (14)	10,955 (11)	**0**.**004**	38 (15)	2,069 (14)	0.56	96 (15)	10,228 (18)	0.02	9 (15)	111 (12)	0.61	94 (13)	5,955 (12)	0.49
Fatigue	455 (55)	46,372 (46)	**<0**.**001**	124 (49)	6,973 (46)	0.33	384 (59)	31,101 (56)	0.09	36 (58)	477 (49)	0.24	367 (51)	19,838 (41)	**<0**.**001**
Cardiovascular diagnostic procedures (%)															
Diagnostic imaging heart	716 (87)	84,102 (83)	**0**.**01**	213 (84)	12,304 (81)	0.18	408 (63)	32,075 (57)	**0**.**007**	32 (52)	469 (49)	0.75	292 (41)	16,206 (33)	**<0**.**001**
Echocardiography	686 (83)	78,733 (78)	**<0**.**001**	204 (81)	11,999 (79)	0.50	385 (59)	30,540 (55)	0.02	31 (50)	449 (47)	0.70	259 (36)	14,363 (29)	**<0**.**001**
Cardiac MRI	53 (6.4)	1,628 (1.6)	**<0**.**001**	54 (21)	793 (5.2)	**<0**.**001**	25 (3.8)	415 (0.74)	**<0**.**001**	6 (9.7)	21 (2.2)	**0**.**002**	3 (0.42)	70 (0.14)	0.16

DCM, dilated cardiomyopathy; HCM, hypertrophic cardiomyopathy; LQTS, long QT syndrome; FH, familial hypercholesterolemia; *N*, number of individuals; n/a, not applicable; IQR, interquartile range; CVD, cardiovascular disease; MRI magnetic resonance imaging.

*P* values in bold type indicate statistical significance after correcting for multiple testing.

^a^ *P* values associated with sex assigned at birth compare males tested to females tested.

For the DCM, LQTS and FH subcohorts, patients receiving genetic testing were significantly more likely to be female (*P *< 0.001 for DCM and LQTS; *P *= 0.005 for FH). Except for those with hereditary amyloidosis, patients receiving genetic testing were significantly more likely to have a family history of CVD (*P *< 0.001 for DCM, HCM, and FH; *P *= 0.003 for LQTS), and for the HCM and LQTS subcohorts also a family history of cardiac arrest (*P *< 0.001 for both subcohorts). In terms of clinical presentation, untested individuals were significantly more likely to also be affected by one or more baseline comorbidities (Table 2). Coronary artery disease was more prevalent in the untested DCM group, possibly correlated to a more advanced age relative to the DCM tested group, but no differences were noted in other indications. Hypertension and hyperlipidemia were more prevalent in all untested groups, except for the FH subcohort where every individual presented with hyperlipidemia as would be expected by clinical diagnostic criteria.

Individuals in the DCM and FH subcohorts who received genetic testing were significantly more likely to have documented cardiovascular symptoms at the time of diagnosis compared with those who did not receive genetic testing. Heartbeat abnormality, chest pain, shortness of breath/dyspnea, syncope/collapse, and fatigue showed statistically significant associations with genetic testing in both subcohorts, while cardiac murmur and abnormal blood pressure were also significant in the DCM cohort (Table 2).

A difference in the utilization of resources was also observed among the subcohorts. Except for those with an FH diagnosis, individuals who received genetic testing were significantly more likely to have documented cardiac magnetic resonance imaging (MRI). Patients with DCM, LQTS, and FH who received genetic testing were significantly more likely to have documented diagnostic imaging of the heart.

**Figure 2:**
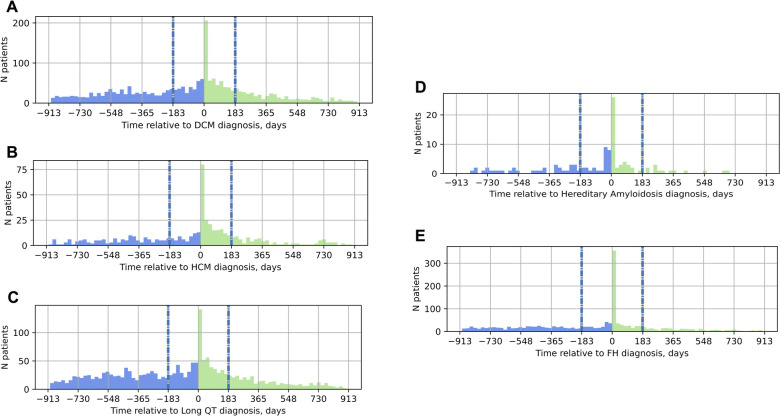
Timing of genetic testing relative to the date of diagnosis in the EHR. On the x-axis, 0 marks the date of diagnosis; the vertical gray and blue dashed lines at −183 days and 183 days mark the six-month window around the date of diagnosis. Panel (**A**) DCM; (**B**) HCM; (**C**) LQTS; (**D**) hereditary amyloidosis; (**E**) FH. Blue bars in the histograms indicate genetic testing before the date of diagnosis, green bars, genetic testing on or after the date of diagnosis. DCM, dilated cardiomyopathy; HCM, hypertrophic cardiomyopathy; LQTS, long QT syndrome; FH, familial hypercholesterolemia.

To understand the role of genetic testing in the clinical care pathway of patients with suspected inherited cardiovascular conditions, the timing of genetic testing relative to the date of diagnosis was also explored (Figure 2). Most individuals received genetic testing on or after their date of diagnosis, not as part of the diagnostic workup; moreover, the most common date of genetic testing across all conditions was the date of diagnosis itself. Of patients diagnosed with DCM, 77% received genetic testing on or after their date of diagnosis. Similarly, 65% of patients diagnosed with HCM, 64% of those diagnosed with LQTS, 66% of those with hereditary amyloidosis, and 74% of those with FH received genetic testing on or after the date of diagnosis.

Further information about the study cohort, including additional comorbidities, and cardiovascular interventions, medications, and laboratory results is provided in Supplementary Table 4. The geographic distribution of the study cohort is shown in Supplementary Figure 1.

## Discussion

This study quantified the current use of guideline-directed genetic testing for 224,641 patients newly diagnosed with DCM, HCM, LQTS, hereditary amyloidosis, or FH in the United States. The profound underutilization of genetic testing observed in this cohort represents a missed opportunity in the evaluation and care of patients with inherited cardiovascular disorders.

Previous multi-center studies have examined adoption of genetic testing in cardiomyopathies and LQTS, reporting adherence ranging from 18% to 92% (50, 51). However, many of those studies were performed on much smaller datasets and were primarily limited to academic medical centers, which do not represent the totality of care provided in the United States. Charron, et al. reported genetic testing for 17.9% and 46.4% of adult patients (*N* = 2,999) with DCM and HCM, respectively, from the Cardiomyopathy Registry of the EURObservational Research Programme consisting of 69 cardiology centers across 18 European countries (50); and Knight, et al. reported genetic testing for 65% and 92% of patients (*N* = 315) with HCM and LQTS, respectively, from six specialty cardiology clinics in large US pediatric healthcare centers (51). The CASCADE-FH registry, however, reports only 3.9% of patients receiving a genetic test (52). In contrast, the current study addressed the utilization of genetic testing in routine clinical practice across the US.

The proportion of patients newly diagnosed with DCM, HCM, LQTS, and FH who received genetic testing in this study ranged from 0.8% to 1.6%. Patients receiving genetic testing in our study were significantly more likely to have a family history of cardiac arrest or cardiovascular disease. Although the number of patients tested is quite low, it is plausible that a positive family history, which in certain situations may signal a potential genetic etiology, could have influenced the use of genetic testing. A positive family history is considered a deciding factor in some guidelines to offer genetic testing; however, family history may not always be available to the clinician or may not be obvious in pedigrees with recessive or X-linked inheritance pattern. Genetic etiology should not be discounted in individuals with negative family history.

Notably, the utilization of genetic testing in 6% of patients diagnosed with hereditary amyloidosis was much higher than for the other subcohorts in this study. While the data do not support specific conclusions, the availability of targeted medications for patients with *TTR* variants (5, 33, 36) may have contributed to the higher utilization of genetic testing in this group.

In the 12 years since publication of the first guideline recommending genetic testing for cardiovascular conditions, evidence and guidance supporting the use of genetic testing in cardiac care has increased (16, 17, 21, 24, 28–38). As this study demonstrates, however, the mere publication of practice guidelines is not sufficient to realize their widespread adoption. Prior studies have suggested the evidence-to-practice gap for translation of empirical evidence can be as long as 17 years or more (53, 54). Ensuring more rapid translation of genetic testing guidelines into practice requires anticipation of barriers and deployment of strategies to overcome those barriers (54–56). Prior studies have identified multiple barriers to the utilization of genetic testing in clinical practice. Among these are: insufficient knowledge of the disorder among physicians and limited cognizance of the utility of incorporating genetic testing into patient care; lack of awareness of guidelines and how to order genetic tests; and, limited availability of, or reimbursement for, genetic testing and related services (16, 19, 22).

Incorporating guideline-based recommendations into quality improvement efforts through EHR integration at the point of care may offer one strategy to overcome barriers and drive utilization of genetic testing in cardiovascular care (57, 58). In 2022, Mohananey et al. (57) reported increased uptake of genetic testing after prescheduling of genomics e-consults for patients with DCM referred to a heart failure clinic. Other approaches have also been proposed. In 2021, Birnbaum et al. (20) and Ingoe et al. (23) demonstrated increased diagnosis of FH and utilization of genetic testing after system-wide scanning of the EHR to identify patients at high risk; and Soper et al. (59) described a genomic screening program that identified individuals with variants in *TTR*, none of whom had a previous diagnosis of hereditary transthyretin amyloidosis. Adopting implementation science frameworks and practices may also facilitate the uptake of guideline-directed genetic testing. Proposed implementation science strategies include leadership engagement, attention to the structure and clarity of guidelines, and the creation of clinical resources to support implementation of recommendations (54–56).

The strength of this study lies in the scope of information available from a nationwide EHR database linked with insurance claims, which includes broad, longitudinal data on patients seen across diverse locations and mixed provider specialties and practice settings as well as insights on billed services such as tests, procedures, and prescriptions. Limitations of the current study, on the other hand, include a lack of data on the types of genetic tests ordered, the percentage of positive genetic test results, the pathogenic variants identified, and the number of patients who were offered genetic testing but declined. The study did not include an analysis of trend of genetic testing utilization over time. Additionally, EHR and claims data are not primarily collected for research purposes so some data elements, such as family history of genetic conditions and results from prior genetic testing of relatives, may be under-recorded. Similar observations were also made in other studies (41, 43, 44).

The current study did not address the relationship between insurance type and rates of genetic testing. However, the claims database used in the analysis includes a mix of commercial and Medicaid Managed Care coverage in roughly similar proportions, combined with Medicare Advantage and other types of coverage in a smaller fraction of cases, thus offering a good representation of different types of coverage available in the US.

In conclusion, this real-world data analysis using a large, nationwide EHR database linked with insurance claims revealed a substantial underutilization of genetic testing for patients in the United States newly diagnosed with DCM, HCM, LQTS, hereditary amyloidosis, and FH. These care gaps persist despite professional practice guidelines recommending genetic testing and the existence of reimbursement policies from major insurers. The importance of establishing a molecular etiology extends beyond diagnostic confirmation and determining the risk of recurrence, as new targeted therapies become available. Collaboration between professional societies, healthcare systems, healthcare providers, payors, and clinical laboratories will be required to increase the utilization of genetic testing as part of an overall strategy to improve guideline adoption and elevate the standard of care and outcome for patients with heritable cardiovascular disorders**.** Further studies are needed to evaluate possible strategies for the implementation of guideline-directed genetic testing and to establish the relationship between genetic testing and patient outcomes.

## Data Availability

The data analyzed in this study is subject to the following licenses/restrictions: The Veradigm Health Insights Ambulatory EHR Research Database linked with insurance claims data is a proprietary dataset. Methodology and results are provided in the manuscript/supplementary data. Requests to access these datasets should be directed to https://veradigm.com.
