# Supplementary material for: Real-world utilization of guideline-directed genetic testing in inherited cardiovascular diseases
Source: Front Cardiovasc Med. 2023 Oct 17;10:1272433. doi: 10.3389/fcvm.2023.1272433 (PMC10616303; doi:10.3389/fcvm.2023.1272433)
Supplement: Supplementary file 1 [file Table1.docx]

***Supplementary Material***

**Real-World Utilization of Guideline-Directed Genetic Testing in Inherited Cardiovascular Diseases**

**Mauro Longoni*, et al.**

***Correspondence:** Corresponding Author: [mlongoni@illumina.com](mailto:mlongoni@illumina.com)

**Supplementary Table 1. ICD-9-CM and ICD-10-CM codes, CPT codes, and filters used to search the Veradigm Health Insights Ambulatory EHR Research Database linked with insurance claims data.**

|  | **DCM** | **HCM** | **LQTS** | **Hereditary Amyloidosis** | **FH** |
| --- | --- | --- | --- | --- | --- |
| **ICD-9-CM and ICD-10-CM codes used** | | | | | |
|  | I25.5, I42.0 | 425.11, 425.18, I42.1, I42.2 | 426.82, I45.81 | E85.0, E85.1, E85.2 | E78.01 |
| **CPT codes used** | | | | | |
| Evidence of genetic testing | 81228, 81229, 81277, 81281, 81306, 81349, 81355, 81400, 81401, 81402, 81404, 81405, 81406, 81407, 81408, 81410, 81411, 81413, 81414, 81415, 81416, 81417, 81425, 81426, 81427, 81439, 81440, 81443, 88245, 88248, 88249, 88261, 88262, 88263, 88264, 88267, 88269, 88271, 88272, 88273, 88280, 88283, 88285, 88289, 88299, 0010U, 0030U, 0094U, 0209U, 0212U, 0213U, 0214U, 0215U, 0237U, 0260U, 0264U, 0267U, 0286U, S3849, S3861, S3865, S3866 | | | | |
| **Filters** | | | | | |
| Age: [18,85] | + | + | + | + | + |
| Dilated cardiomyopathy, familial: [no] |  | + | + | + | + |
| Dilated cardiomyopathy: [no] |  | + | + | + | + |
| Hypertrophic cardiomyopathy - nonobstructive: [no] | + |  | + | + | + |
| Hypertrophic cardiomyopathy - obstructive: [no] | + |  | + | + | + |
| Hypertrophic cardiomyopathy - unspecified: [no] | + |  | + | + | + |
| Hypertrophic cardiomyopathy (familial): [no] | + |  | + | + | + |
| Long QT syndrome: [no] | + | + |  | + | + |
| Amyloidosis, Hereditary neuropathic: [no] | + | + | + |  | + |
| Amyloidosis, Hereditary non-neuropathic: [no] | + | + | + |  | + |
| Amyloidosis, Hereditary unspecified: [no] | + | + | + |  | + |
| Hereditary amyloidosis: [no] | + | + | + |  | + |
| Familial hypercholesterolemia, heterozygous: [no] | + | + | + | + |  |
| Familial hypercholesterolemia, homozygous: [no] | + | + | + | + |  |
| Familial hypercholesterolemia, unspecified: [no] | + | + | + | + |  |
| Familial hypercholesterolemia: [no] | + | + | + | + |  |
| Cancer: [no] | + | + | + | + | + |
| Cystic fibrosis: [no] | + | + | + | + | + |
| Current pregnancy: [no] | + | + | + | + | + |

Filters omitted from condition-specific searches of the EHR are indicated by shaded cells. Abbreviations: EHR, electronic health record; ICD-9-CM and ICD-10-CM, International Classification of Diseases, Clinical Modification, 9^th^ and 10^th^ revisions, respectively; CPT, Current Procedural Terminology, DCM, dilated cardiomyopathy; HCM, hypertrophic cardiomyopathy; LQTS, long QT syndrome; FH, familial hypercholesterolemia.

**Supplementary Table 2. Guideline and statement genetic testing recommendations codified for analysis, by condition.**

| **Recommendations** | **COR** | **Level of Evidence** |
| --- | --- | --- |
| **Cardiomyopathy, general/other conditions** | | |
| “In first-degree relatives of selected patients with genetic or inherited cardiomyopathies, genetic screening and counseling are recommended to detect cardiac disease and prompt consideration of treatments to decrease HF progression and sudden death.” (1) | 1 | B-NR |
| “In patients with NICM who develop conduction disease or LV dysfunction at less than 40 years of age, or who have a family history of NICM or SCD in a first-degree relative (<50 years of age), genetic counseling and genetic testing are reasonable to detect a heritable disease that may clarify prognosis and facilitate cascade screening of relatives.” (2) | 2a | C-EO |
| “In patients with familial or idiopathic cardiomyopathy, genetic testing can be useful in conjunction with genetic counseling….” (3) | - | B |
|  | | |
| **Dilated cardiomyopathy, specific** | | |
| “Comprehensive or targeted DCM genetic testing (*LMNA* and *SCN5A*) is recommended for patients with DCM and significant cardiac conduction disease (i.e., first-, second-, or third-degree heart block) or a family history of premature unexpected sudden death….” (3) | - | A |
| “Mutation-specific genetic testing is recommended for family members and appropriate relatives after the identification of a DCM-causative mutation in the index case….” (3) | - | B |
| “Genetic testing can be useful for patients with familial DCM to confirm the diagnosis, facilitate cascade screening within the family, and help with family planning….” (3) | - | A |
| “In pediatric patients with DCM phenotype, and musculoskeletal symptoms such as hypotonia, a skeletal muscle biopsy may aid in the diagnosis, and genetic testing may be considered….” (3) | - | C |
| “Genetic testing is recommended for patients with cardiomyopathy. … Cascade genetic testing of at-risk family members is recommended for pathogenic and likely pathogenic variants.” — “Dilated cardiomyopathy” (4) | - | A |
|  | | |
| **Hypertrophic cardiomyopathy, specific** | | |
| “In first-degree relatives of patients with HCM due to a known causative mutation, genetic counseling and mutation-specific genetic testing are recommended.” (2) | 1 | B-NR |
| “In patients with clinically suspected or diagnosed HCM, genetic counseling and genetic testing are reasonable.” (2) | 2a | B-NR |
| “Genetic testing is recommended for patients with cardiomyopathy. … Cascade genetic testing of at-risk family members is recommended for pathogenic and likely pathogenic variants.” — “Hypertrophic cardiomyopathy (HCM)” (4) | - | A |
| “In patients with HCM, genetic testing is beneficial to elucidate the genetic basis to facilitate the identification of family members at risk for developing HCM (cascade testing).” (5) | 1 | B-NR |
| “In patients with an atypical clinical presentation of HCM or when another genetic condition is suspected to be the cause, a work-up including genetic testing for HCM and other genetic causes of unexplained cardiac hypertrophy (“HCM phenocopies”) is recommended.” (5) | 1 | B-NR |
| “When performing genetic testing in an HCM proband, the initial tier of genes tested should include genes with strong evidence to be disease-causing in HCM.” (5) | 1 | B-NR |
| “In first-degree relatives of patients with HCM, both clinical screening (ECG and 2D echocardiogram) and cascade genetic testing (when a pathogenic/likely pathogenic variant has been identified in the proband) should be offered.” (5) | 1 | B-NR |
|  | | |
| **Long QT syndrome** | | |
| “In first-degree relatives of patients who have a causative mutation for long QT syndrome, catecholaminergic polymorphic ventricular tachycardia, short QT syndrome, or Brugada syndrome, genetic counseling and mutation-specific genetic testing are recommended.” (2) | 1 | B-NR |
| “In patients with clinically diagnosed long QT syndrome, genetic counseling and genetic testing are recommended.” (2) | 1 | B-NR |
|  | | |
| **Hereditary amyloidosis** | | |
| “In patients for whom a diagnosis of transthyretin cardiac amyloidosis is made, genetic testing with *TTR* gene sequencing is recommended to differentiate hereditary variant from wild-type transthyretin cardiac amyloidosis.” (1) | 1 | B-NR |
|  | | |
| **Familial hypercholesterolemia** | | |
| “In children and adolescents found to have moderate or severe hypercholesterolemia, it is reasonable to carry out reverse-cascade screening of family members, which includes cholesterol testing for first-, second-, and when possible, third-degree biological relatives, for detection of familial forms of hypercholesterolemia.” (6) | 2a | B-NR |
| “Genetic testing for FH may be considered in the following clinical scenarios: 1. Children with persistent LDL-C levels ≥160 mg/dl (without an apparent secondary cause of hypercholesterolemia) with an LDL-C Level ≥190 mg/dl in at least 1 parent or a family history of hypercholesterolemia and premature CAD” (7) | 2b | C-EO |
| “Genetic testing for FH may be considered in the following clinical scenarios: … 2. Adults with no pre-treatment LDL-C levels available but with a personal history of premature CAD and family history of both hypercholesterolemia and premature CAD” (7) | 2b | C-EO |
| “Genetic testing for FH may be considered in the following clinical scenarios: … 3. Adults with persistent LDL-C levels ≥160 mg/dl (without an apparent secondary cause of hypercholesterolemia) in the setting of a family history of hypercholesterolemia and either a personal history or a family history of premature CAD” (7) | 2b | C-EO |
| “Cascade genetic testing for the specific variant(s) identified in the FH proband (known familial variant testing) should be offered to all first-degree relatives. If first-degree relatives are unavailable, or do not wish to undergo testing, known familial variant testing should be offered to second-degree relatives. Cascade genetic testing should commence throughout the entire extended family until all at-risk individuals have been tested and all known relatives with FH have been identified” (7) | 1 | B-R |
| “Genetic testing for FH should be offered to individuals of any age in whom a strong clinical index of suspicion for FH exists based on examination of the patient’s clinical and/or family histories. This index of suspicion includes the following: 1. Children with persistent LDL-C levels ≥160 mg/dl or adults with persistent LDL-C levels ≥190 mg/dl without an apparent secondary cause of hypercholesterolemia and with at least 1 first-degree relative similarly affected or with premature CAD or where family history is not available (e.g., adoption)” (7) | 2a | B-NR |
| “Genetic testing for FH should be offered to individuals of any age in whom a strong clinical index of suspicion for FH exists based on examination of the patient’s clinical and/or family histories. This index of suspicion includes the following: … 2. Children with persistent LDL-C levels ≥190 mg/dl or adults with persistent LDL-C levels ≥250 mg/dl without an apparent secondary cause of hypercholesterolemia, even in the absence of a positive family history” (7) | 2a | B-NR |

Abbreviations: COR, class of recommendation; B-R, level B randomized; B-NR, level B non-randomized; C-EO, level C expert opinion; -, not classified according to numeric COR or level of evidence; level A, strong evidence; level B moderate evidence; level C, limited evidence/expert opinion; HF, heart failure; NICM, non-ischemic cardiomyopathy; LV, left ventricular; SCD, sudden cardiac death; DCM, dilated cardiomyopathy; HCM, hypertrophic cardiomyopathy; ECG, electrocardiogram; 2D, two-dimensional; FH, familial hypercholesterolemia; LDL-C, low-density lipoprotein-cholesterol; mg/dl, milligrams per deciliter; CAD, coronary artery disease.

**Supplementary Table 3. Guideline and statement genetic testing recommendations codified for analysis, by guideline/statement**

| **Guideline/Statement** | **Recommendation** | **COR** | **Level of Evidence** |
| --- | --- | --- | --- |
| 2022 AHA/ACC/HFSA Guideline for the Management of Heart Failure: A Report of the American College of Cardiology/American Heart Association Joint Committee on Clinical Practice Guidelines (1) | “In first-degree relatives of selected patients with genetic or inherited cardiomyopathies, genetic screening and counseling are recommended to detect cardiac disease and prompt consideration of treatments to decrease HF progression and sudden death.” | 1 | B-NR |
|  | “In patients for whom a diagnosis of transthyretin cardiac amyloidosis is made, genetic testing with *TTR* gene sequencing is recommended to differentiate hereditary variant from wild-type transthyretin cardiac amyloidosis.” | 1 | B-NR |
| 2020 AHA/ACC Guideline for the Diagnosis and Treatment of Patients With Hypertrophic Cardiomyopathy: Executive Summary: A Report of the American College of Cardiology/American Heart Association Joint Committee on Clinical Practice Guidelines (5) | “In patients with HCM, genetic testing is beneficial to elucidate the genetic basis to facilitate the identification of family members at risk for developing HCM (cascade testing).” | 1 | B-NR |
|  | “In patients with an atypical clinical presentation of HCM or when another genetic condition is suspected to be the cause, a work-up including genetic testing for HCM and other genetic causes of unexplained cardiac hypertrophy (“HCM phenocopies”) is recommended.” | 1 | B-NR |
|  | “When performing genetic testing in an HCM proband, the initial tier of genes tested should include genes with strong evidence to be disease-causing in HCM.” | 1 | B-NR |
|  | “In first-degree relatives of patients with HCM, both clinical screening (ECG and 2D echocardiogram) and cascade genetic testing (when a pathogenic/likely pathogenic variant has been identified in the proband) should be offered.” | 1 | B-NR |
| 2018 AHA/ACC/AACVPR/AAPA/ABC/ ACPM/ADA/AGS/APhA/ASPC/NLA/PCNA Guideline on the Management of Blood Cholesterol: A Report of the American College of Cardiology/American Heart Association Task Force on Clinical Practice Guidelines (6) | “In children and adolescents found to have moderate or severe hypercholesterolemia, it is reasonable to carry out reverse-cascade screening of family members, which includes cholesterol testing for first-, second-, and when possible, third-degree biological relatives, for detection of familial forms of hypercholesterolemia.” | 2a | B-NR |
| (2018) Clinical Genetic Testing for Familial Hypercholesterolemia: JACC Scientific Expert Panel (7) | “Genetic testing for FH may be considered in the following clinical scenarios: 1. Children with persistent LDL-C levels ≥160 mg/dl (without an apparent secondary cause of hypercholesterolemia) with an LDL-C Level ≥190 mg/dl in at least 1 parent or a family history of hypercholesterolemia and premature CAD” | 2b | C-EO |
|  | “Genetic testing for FH may be considered in the following clinical scenarios: … 2. Adults with no pre-treatment LDL-C levels available but with a personal history of premature CAD and family history of both hypercholesterolemia and premature CAD” | 2b | C-EO |
|  | “Genetic testing for FH may be considered in the following clinical scenarios: … 3. Adults with persistent LDL-C levels ≥160 mg/dl (without an apparent secondary cause of hypercholesterolemia) in the setting of a family history of hypercholesterolemia and either a personal history or a family history of premature CAD” | 2b | C-EO |
|  | “Cascade genetic testing for the specific variant(s) identified in the FH proband (known familial variant testing) should be offered to all first-degree relatives. If first-degree relatives are unavailable, or do not wish to undergo testing, known familial variant testing should be offered to second-degree relatives. Cascade genetic testing should commence throughout the entire extended family until all at-risk individuals have been tested and all known relatives with FH have been identified” | 1 | B-R |
|  | “Genetic testing for FH should be offered to individuals of any age in whom a strong clinical index of suspicion for FH exists based on examination of the patient’s clinical and/or family histories. This index of suspicion includes the following: 1. Children with persistent LDL-C levels ≥160 mg/dl or adults with persistent LDL-C levels ≥190 mg/dl without an apparent secondary cause of hypercholesterolemia and with at least 1 first-degree relative similarly affected or with premature CAD or where family history is not available (e.g., adoption)” | 2a | B-NR |
|  | “Genetic testing for FH should be offered to individuals of any age in whom a strong clinical index of suspicion for FH exists based on examination of the patient’s clinical and/or family histories. This index of suspicion includes the following: … 2. Children with persistent LDL-C levels ≥190 mg/dl or adults with persistent LDL-C levels ≥250 mg/dl without an apparent secondary cause of hypercholesterolemia, even in the absence of a positive family history” | 2a | B-NR |
| (2018) Genetic Evaluation of Cardiomyopathy—A Heart Failure Society of America Practice Guideline (4) | “Genetic testing is recommended for patients with cardiomyopathy. … Cascade genetic testing of at-risk family members is recommended for pathogenic and likely pathogenic variants.” | - | - |
|  | “Hypertrophic cardiomyopathy (HCM)” | - | A |
|  | “Dilated cardiomyopathy (DCM)” | - | A |
| 2017 AHA/ACC/HRS Guideline for Management of Patients With Ventricular Arrhythmias and the Prevention of Sudden Cardiac Death: A Report of the American College of Cardiology/American Heart Association Task Force on Clinical Practice Guidelines and the Heart Rhythm Society (2) | “In patients with NICM who develop conduction disease or LV dysfunction at less than 40 years of age, or who have a family history of NICM or SCD in a first-degree relative (<50 years of age), genetic counseling and genetic testing are reasonable to detect a heritable disease that may clarify prognosis and facilitate cascade screening of relatives.” | 2a | C-EO |
|  | “In first-degree relatives of patients with HCM due to a known causative mutation, genetic counseling and mutation-specific genetic testing are recommended.” | 1 | B-NR |
|  | “In patients with clinically suspected or diagnosed HCM, genetic counseling and genetic testing are reasonable.” | 2a | B-NR |
|  | “In first-degree relatives of patients who have a causative mutation for long QT syndrome, catecholaminergic polymorphic ventricular tachycardia, short QT syndrome, or Brugada syndrome, genetic counseling and mutation-specific genetic testing are recommended.” | 1 | B-NR |
|  | “In patients with clinically diagnosed long QT syndrome, genetic counseling and genetic testing are recommended.” | 1 | B-NR |
| (2016) Current Diagnostic and Treatment Strategies for Specific Dilated Cardiomyopathies: A Scientific Statement From the American Heart Association (3) | “Comprehensive or targeted DCM genetic testing (*LMNA* and *SCN5A*) is recommended for patients with DCM and significant cardiac conduction disease (i.e., first-, second-, or third-degree heart block) or a family history of premature unexpected sudden death….” | - | A |
|  | “Mutation-specific genetic testing is recommended for family members and appropriate relatives after the identification of a DCM-causative mutation in the index case….” | - | B |
|  | “Genetic testing can be useful for patients with familial DCM to confirm the diagnosis, facilitate cascade screening within the family, and help with family planning….” | - | A |
|  | “In pediatric patients with DCM phenotype, and musculoskeletal symptoms such as hypotonia, a skeletal muscle biopsy may aid in the diagnosis, and genetic testing may be considered….” | - | C |
|  | “In patients with familial or idiopathic cardiomyopathy, genetic testing can be useful in conjunction with genetic counseling….” | - | B |

Abbreviations: COR, class of recommendation; B-R, level B randomized; B-NR, level B non-randomized; C-EO, level C expert opinion; -, not classified according to numeric COR or level of evidence; level A, strong evidence; level B moderate evidence; level C, limited evidence/expert opinion; ACC, American College of Cardiology; AHA, American Heart Association; HFSA, Heart Failure Society of America; AACVPR, American Association of Cardiovascular and Pulmonary Rehabilitation; AAPA, American Academy of Physician Assistants; ABC, Association of Black Cardiologists; ACPM, American College of Preventive Medicine; ADA, American Diabetes Association; AGS, American Geriatrics Society; APhA, American Pharmacists Association; ASPC, American Society for Preventive Cardiology; NLA, National Lipid Association; PCNA, Preventive Cardiovascular Nurses Association; JACC, Journal of the American College of Cardiology; HRS, Heart Rhythm Society; HF, heart failure; HCM, hypertrophic cardiomyopathy; ECG, electrocardiogram; 2D, two-dimensional; FH, familial hypercholesterolemia; LDL-C, low-density lipoprotein-cholesterol; mg/dl, milligrams per deciliter; CAD, coronary artery disease; DCM, dilated cardiomyopathy; NICM, non-ischemic cardiomyopathy; LV, left ventricular; SCD, sudden cardiac death.

**Supplementary Table 4. Additional baseline comorbidities, cardiovascular intervention procedures, cardiovascular medications, and cardiovascular laboratory results of the study cohort, by condition and evidence of genetic testing**

|  | **DCM**  **N = 101,919** | | | **HCM**  **N = 15,507** | | | **LQTS**  **N = 56,539** | | | **Hereditary Amyloidosis**  **N = 1,026** | | | **FH**  **N = 49,650** | | |  |
| --- | --- | --- | --- | --- | --- | --- | --- | --- | --- | --- | --- | --- | --- | --- | --- | --- |
| **Evidence of genetic testing (+/-), *P* value** | + | - | *P* | + | - | *P* | + | - | *P* | + | - | *P* | + | - | *P* |  |
| **N** | 827 | 101,092 | n/a | 253 | 15,254 | n/a | 650 | 55,889 | n/a | 62 | 964 | n/a | 718 | 48,932 | n/a |  |
| **Additional baseline comorbidities (%, if > 1%)** | | | | | | | | | | | | | | | | |
| Heart failure | 561 (68) | 72,837 (72) | 0.008 | 73 (29) | 5,303 (35) | 0.06 | 180 (28) | 18,020 (32) | 0.02 | 9 (15) | 162 (17) | 0.77 | 92 (13) | 3,803 (7.8) | <.001 |  |
| Long QT syndrome | 0 | 0 | 1.0 | 0 | 0 | 1.0 | 650 (100) | 55,889 (100) | 1.0 | 0 | 0 | 1.0 | 0 | 0 | 1.0 |  |
| Ventricular arrhythmia | 129 (16) | 14,428 (14) | 0.3 | 19 (7.5) | 860 (5.6) | 0.25 | 80 (12) | 3,321 (5.9) | <.001 | 4 (6.5) | 22 (2.3) | 0.11 | 15 (2.1) | 497 (1) | 0.008 |  |
| Amyloidosis | 15 (1.8) | 316 | <.001 | 7 (2.8) | 79 | <.001 | 3 | 167 | 0.69 | 62 (100) | 964 (100) | 1.0 | 2 | 70 | 0.65 |  |
| Hereditary amyloidosis | 0 | 0 | 1.0 | 0 | 0 | 1.0 | 0 | 0 | 1.0 | 62 (100) | 964 (100) | 1.0 | 0 | 0 | 1.0 |  |
| Cardiomyopathy | 827 (100) | 101,092 (100) | 1.0 | 253 (100) | 15,254 (100) | 1.0 | 69 (11) | 4,649 (8.3) | 0.04 | 10 (16) | 51 (5.3) | 0.001 | 14 (1.9) | 905 (1.8) | 0.95 |  |
| Dilated cardiomyopathy | 827 (100) | 101,092 (100) | 1.0 | 0 | 0 | 1.0 | 0 | 0 | 1.0 | 0 | 0 | 1.0 | 0 | 0 | 1.0 |  |
| Familial hypercholesterolemia | 0 | 0 | 1.0 | 0 | 0 | 1.0 | 0 | 0 | 1.0 | 0 | 0 | 1.0 | 718 (100) | 48,932 (100) | 1.0 |  |
| Cancer | 0 | 0 | 1.0 | 0 | 0 | 1.0 | 0 | 0 | 1.0 | 0 | 0 | 1.0 | 0 | 0 | 1.0 |  |
| Angioedema | 6 | 795 | 1.0 | 2 | 126 | 1.0 | 15 (2.3) | 693 (1.2) | 0.02 | 1 (1.6) | 10 (1) | 1.0 | 6 | 305 | 0.63 |  |
| Aortic valve stenosis | 60 (7.3) | 7,420 (7.3) | 0.98 | 27 (11) | 1,335 (8.8) | 0.34 | 19 (2.9) | 2,549 (4.6) | 0.06 | 1 (1.6) | 26 (2.7) | 0.91 | 12 (1.7) | 891 (1.8) | 0.88 |  |
| Atrial fibrillation | 241 (29) | 32,882 (33) | 0.04 | 39 (15) | 2,760 (18) | 0.31 | 102 (16) | 11,009 (20) | 0.01 | 1 (1.6) | 96 (10) | 0.05 | 43 (6) | 2,784 (5.7) | 0.79 |  |
| Cardiac arrest | 51 (6.2) | 5,086 (5) | 0.16 | 10 (4) | 320 (2.1) | 0.07 | 31 (4.8) | 2,336 (4.2) | 0.52 | 0 | 7 | 1.0 | 4 | 162 | 0.47 |  |
| HFpEF | 189 (23) | 24,890 (25) | 0.26 | 51 (20) | 3,079 (20) | 1.0 | 95 (15) | 9,711 (17) | 0.07 | 9 (15) | 79 (8.2) | 0.14 | 35 (4.9) | 1,650 (3.4) | 0.04 |  |
| HFrEF | 389 (47) | 50,685 (50) | 0.08 | 16 (6.3) | 1,508 (9.9) | 0.07 | 69 (11) | 6,632 (12) | 0.36 | 5 (8.1) | 52 (5.4) | 0.55 | 17 (2.4) | 870 (1.8) | 0.3 |  |
| Hemochromatosis,  hereditary | 5 | 125 | <.001 | 3 (1.2) | 23 | 0.001 | 7 (1.1) | 86 | <.001 | 0 | 2 | 1.0 | 6 | 71 | <.001 |  |
| Hypertrophic cardiomyopathy (familial) | 0 | 0 | 1.0 | 1 | 11 | 0.49 | 0 | 0 | 1.0 | 0 | 0 | 1.0 | 0 | 0 | 1.0 |  |
| Hypertrophic cardiomyopathy –nonobstructive | 0 | 0 | 1.0 | 2 | 14 | 0.01 | 0 | 0 | 1.0 | 0 | 0 | 1.0 | 0 | 0 | 1.0 |  |
| Hypertrophic cardiomyopathy – obstructive | 0 | 0 | 1.0 | 63 (25) | 3,728 (24) | 0.92 | 0 | 0 | 1.0 | 0 | 0 | 1.0 | 0 | 0 | 1.0 |  |
| Hypertrophic cardiomyopathy – unspecified | 0 | 0 | 1.0 | 188 (74) | 11,512 (75) | 0.73 | 0 | 0 | 1.0 | 0 | 0 | 1.0 | 0 | 0 | 1.0 |  |
| Liver disease | 230 (28) | 20,366 (20) | <.001 | 52 (21) | 3,052 (20) | 0.89 | 190 (29) | 18,290 (33) | 0.06 | 12 (19) | 241 (25) | 0.4 | 146 (20) | 7,818 (16) | 0.002 |  |
| Obesity | 427 (52) | 53,566 (53) | 0.46 | 126 (50) | 8,153 (53) | 0.28 | 311 (48) | 28,626 (51) | 0.09 | 24 (39) | 454 (47) | 0.25 | 315 (44) | 21,017 (43) | 0.65 |  |
| Smoking history | 4 | 306 | 0.53 | 0 | 55 | 0.67 | 1 | 161 | 0.79 | 0 | 1 | 1.0 | 1 | 102 | 1.0 |  |
| Pulmonary hypertension, primary | 19 (2.3) | 2,668 (2.6) | 0.62 | 6 (2.4) | 373 (2.4) | 1.0 | 9 (1.4) | 1,210 (2.2) | 0.22 | 2 (3.2) | 15 (1.6) | 0.63 | 5 | 232 | 0.56 |  |
| Renal disease | 428 (52) | 50,372 (50) | 0.29 | 81 (32) | 5,857 (38) | 0.04 | 249 (38) | 25,667 (46) | <.001 | 18 (29) | 330 (34) | 0.48 | 140 (19) | 8,859 (18) | 0.36 |  |
| Hemorrhagic stroke | 241 (29) | 28,780 (28) | 0.7 | 49 (19) | 3,769 (25) | 0.06 | 141 (22) | 14,504 (26) | 0.02 | 10 (16) | 231 (24) | 0.21 | 101 (14) | 5,956 (12) | 0.14 |  |
| Ischemic stroke | 130 (16) | 14,786 (15) | 0.4 | 25 (9.9) | 1,884 (12) | 0.28 | 78 (12) | 8,448 (15) | 0.03 | 5 (8.1) | 102 (11) | 0.68 | 44 (6.1) | 2,491 (5.1) | 0.24 |  |
| TIA | 58 (7) | 6,264 (6.2) | 0.37 | 13 (5.1) | 909 (6) | 0.68 | 35 (5.4) | 3,481 (6.2) | 0.42 | 3 (4.8) | 50 (5.2) | 1.0 | 26 (3.6) | 1,519 (3.1) | 0.49 |  |
| Ventricular fibrillation | 33 (4) | 2,840 (2.8) | 0.05 | 5 (2) | 101 | 0.03 | 31 (4.8) | 730 (1.3) | <.001 | 0 | 1 | 1.0 | 4 | 74 | 0.02 |  |
| Ventricular tachycardia | 116 (14) | 13,061 (13) | 0.37 | 18 (7.1) | 807 (5.3) | 0.25 | 67 (10) | 2,944 (5.3) | <.001 | 4 (6.5) | 21 (2.2) | 0.09 | 13 (1.8) | 450 | 0.02 |  |
| Dilated cardiomyopathy, familial | 0 | 3 | 1.0 | 0 | 0 | 1.0 | 0 | 0 | 1.0 | 0 | 0 | 1.0 | 0 | 0 | 1.0 |  |
| Amyloidosis, neuropathic | 0 | 1 | 1.0 | 0 | 0 | 1.0 | 0 | 0 | 1.0 | 0 | 3 | 1.0 | 0 | 0 | 1.0 |  |
| Amyloidosis, hereditary neuropathic | 0 | 0 | 1.0 | 0 | 0 | 1.0 | 0 | 0 | 1.0 | 18 (29) | 380 (39) | 0.14 | 0 | 0 | 1.0 |  |
| Amyloidosis, hereditary non-neuropathic | 0 | 0 | 1.0 | 0 | 0 | 1.0 | 0 | 0 | 1.0 | 30 (48) | 431 (45) | 0.67 | 0 | 0 | 1.0 |  |
| Amyloidosis, hereditary unspecified | 0 | 0 | 1.0 | 0 | 0 | 1.0 | 0 | 0 | 1.0 | 15 (24) | 161 (17) | 0.18 | 0 | 0 | 1.0 |  |
| Amyloidosis, organ limited | 9 (1.1) | 143 | <.001 | 3 (1.2) | 37 | 0.02 | 2 | 89 | 0.66 | 9 (15) | 47 (4.9) | 0.003 | 1 | 17 | 0.64 |  |
| Amyloidosis, other | 4 | 56 | <.001 | 3 (1.2) | 14 | <.001 | 1 | 38 | 0.94 | 13 (21) | 48 (5) | <.001 | 1 | 7 | 0.26 |  |
| Amyloidosis, secondary systemic | 0 | 5 | 1.0 | 0 | 0 | 1.0 | 1 | 18 | 0.54 | 0 | 3 | 1.0 | 0 | 4 | 1.0 |  |
| Amyloidosis, unspecified | 9 (1.1) | 116 | <.001 | 6 (2.4) | 34 | <.001 | 0 | 58 | 0.84 | 13 (21) | 70 (7.3) | <.001 | 0 | 19 | 1.0 |  |
| Cardiomyopathy, alcoholic | 11 (1.3) | 1,040 (1) | 0.5 | 0 | 20 | 1.0 | 1 | 160 | 0.8 | 0 | 1 | 1.0 | 0 | 11 | 1.0 |  |
| Cardiomyopathy, drug/external agent | 3 | 918 | 0.14 | 0 | 28 | 1.0 | 1 | 111 | 1.0 | 0 | 0 | 1.0 | 0 | 3 | 1.0 |  |
| Cardiomyopathy, other | 196 (24) | 21,174 (21) | 0.06 | 28 (11) | 1,032 (6.8) | 0.01 | 31 (4.8) | 1,963 (3.5) | 0.11 | 4 (6.5) | 21 (2.2) | 0.09 | 4 | 389 | 0.62 |  |
| Cardiomyopathy unspecified | 316 (38) | 34,867 (34) | 0.03 | 41 (16) | 1,878 (12) | 0.08 | 55 (8.5) | 3,842 (6.9) | 0.13 | 10 (16) | 46 (4.8) | <.001 | 13 (1.8) | 729 (1.5) | 0.58 |  |
| Chronic kidney disease | 351 (42) | 40,670 (40) | 0.21 | 68 (27) | 4,696 (31) | 0.2 | 169 (26) | 17,493 (31) | 0.004 | 15 (24) | 257 (27) | 0.78 | 103 (14) | 6,667 (14) | 0.61 |  |
| End stage renal disease | 77 (9.3) | 7,421 (7.3) | 0.04 | 11 (4.3) | 688 (4.5) | 1.0 | 42 (6.5) | 5,329 (9.5) | 0.01 | 3 (4.8) | 34 (3.5) | 0.85 | 3 | 479 | 0.18 |  |
| Heart failure, ACC/AHA stage B | 0 | 11 | 1.0 | 0 | 1 | 1.0 | 0 | 1 | 1.0 | 0 | 0 | 1.0 | 0 | 0 | 1.0 |  |
| Heart failure, ACC/AHA stage C | 1 | 105 | 1.0 | 0 | 4 | 1.0 | 0 | 15 | 1.0 | 0 | 0 | 1.0 | 0 | 4 | 1.0 |  |
| Heart failure, ACC/AHA stage D | 0 | 0 | 1.0 | 0 | 0 | 1.0 | 0 | 0 | 1.0 | 0 | 0 | 1.0 | 0 | 0 | 1.0 |  |
| Heart failure, NYHA class 1 | 1 | 64 | 1.0 | 0 | 4 | 1.0 | 0 | 10 | 1.0 | 0 | 0 | 1.0 | 0 | 5 | 1.0 |  |
| Heart failure, NYHA class 2 | 2 | 193 | 1.0 | 0 | 12 | 1.0 | 0 | 40 | 1.0 | 0 | 0 | 1.0 | 0 | 4 | 1.0 |  |
| Heart failure, NYHA class 3 | 4 | 207 | 0.17 | 1 | 9 | 0.4 | 0 | 41 | 1.0 | 0 | 1 | 1.0 | 0 | 5 | 1.0 |  |
| Heart failure, NYHA class 4 | 0 | 5 | 1.0 | 0 | 0 | 1.0 | 0 | 0 | 1.0 | 0 | 0 | 1.0 | 0 | 0 | 1.0 |  |
| Heart failure, other | 35 (4.2) | 4,091 (4) | 0.86 | 1 | 233 (1.5) | 0.23 | 21 (3.2) | 1,183 (2.1) | 0.07 | 0 | 6 | 1.0 | 5 | 88 | 0.006 |  |
| Heart failure, rheumatic | 0 | 95 | 0.76 | 0 | 7 | 1.0 | 0 | 29 | 1.0 | 0 | 0 | 1.0 | 0 | 5 | 1.0 |  |
| Heart failure, systolic and diastolic | 166 (20) | 21,060 (21) | 0.62 | 6 (2.4) | 693 (4.5) | 0.13 | 28 (4.3) | 3,054 (5.5) | 0.23 | 2 (3.2) | 23 (2.4) | 1.0 | 3 | 379 | 0.38 |  |
| Familial hypercholesterolemia, heterozygous | 0 | 0 | 1.0 | 0 | 0 | 1.0 | 0 | 0 | 1.0 | 0 | 0 | 1.0 | 2 | 64 | 0.57 |  |
| Familial hypercholesterolemia, homozygous | 0 | 0 | 1.0 | 0 | 0 | 1.0 | 0 | 0 | 1.0 | 0 | 0 | 1.0 | 1 | 14 | 0.54 |  |
| Familial hypercholesterolemia, unspecified | 0 | 0 | 1.0 | 0 | 0 | 1.0 | 0 | 0 | 1.0 | 0 | 0 | 1.0 | 16 (2.2) | 1,767 (3.6) | 0.06 |  |
| Metabolic syndrome | 91 (11) | 12,395 (12) | 0.3 | 29 (11) | 1,791 (12) | 0.97 | 76 (12) | 5,626 (10) | 0.19 | 2 (3.2) | 89 (9.2) | 0.17 | 88 (12) | 5,417 (11) | 0.34 |  |
| **Cardiovascular intervention procedures (%, if > 1%)** | | | | | | | | | | | | | | | | |
| Angioplasty, cardiac | 94 (11) | 14,892 (15) | 0.008 | 4 (1.6) | 458 (3) | 0.26 | 19 (2.9) | 1,841 (3.3) | 0.68 | 1 (1.6) | 10 (1) | 1.0 | 19 (2.6) | 942 (1.9) | 0.21 |  |
| Angioplasty, peripheral | 24 (2.9) | 2,906 (2.9) | 1.0 | 2 | 159 (1) | 0.94 | 6 | 840 (1.5) | 0.29 | 0 | 12 (1.2) | 0.78 | 4 | 305 | 1.0 |  |
| Artery stent, cardiac | 94 (11) | 14,396 (14) | 0.02 | 4 (1.6) | 439 (2.9) | 0.3 | 19 (2.9) | 1,779 (3.2) | 0.79 | 1 (1.6) | 10 (1) | 1.0 | 19 (2.6) | 916 (1.9) | 0.17 |  |
| Artery stent, peripheral | 11 (1.3) | 1,450 (1.4) | 0.92 | 1 | 71 | 1.0 | 5 | 414 | 1.0 | 0 | 2 | 1.0 | 4 | 165 | 0.5 |  |
| Cardiac resynchronization therapy | 14 (1.7) | 1,227 (1.2) | 0.27 | 0 | 19 | 1.0 | 1 | 40 | 0.97 | 0 | 1 | 1.0 | 0 | 16 | 1.0 |  |
| Implantable cardiac defibrillator | 117 (14) | 14,479 (14) | 0.93 | 14 (5.5) | 711 (4.7) | 0.62 | 37 (5.7) | 2,382 (4.3) | 0.09 | 1 (1.6) | 17 (1.8) | 1.0 | 17 (2.4) | 513 (1) | 0.001 |  |
| Pacemaker | 42 (5.1) | 4,923 (4.9) | 0.84 | 6 (2.4) | 234 (1.5) | 0.42 | 14 (2.2) | 664 (1.2) | 0.04 | 1 (1.6) | 9 | 1.0 | 4 | 189 | 0.67 |  |
| Percutaneous coronary intervention | 1 | 46 | 0.85 | 0 | 5 | 1.0 | 0 | 10 | 1.0 | 0 | 0 | 1.0 | 0 | 1 | 1.0 |  |
| Revascularization, cardiac | 49 (5.9) | 6,617 (6.5) | 0.52 | 0 | 102 | 0.36 | 4 | 537 | 0.49 | 0 | 3 | 1.0 | 5 | 247 | 0.65 |  |
| Revascularization, peripheral | 23 (2.8) | 2,856 (2.8) | 1.0 | 1 | 156 (1) | 0.5 | 6 | 811 (1.5) | 0.34 | 0 | 11 (1.1) | 0.83 | 4 | 303 | 1.0 |  |
| EP study with ablation | 18 (2.2) | 1,905 (1.9) | 0.63 | 6 (2.4) | 184 (1.2) | 0.17 | 13 (2) | 1,014 (1.8) | 0.84 | 0 | 5 | 1.0 | 5 | 161 | 0.17 |  |
| Artery stent, other | 5 | 769 | 0.75 | 0 | 63 | 0.6 | 4 | 496 | 0.6 | 0 | 1 | 1.0 | 2 | 77 | 0.74 |  |
| **Cardiovascular medications (%, if > 1%)** | | | | | | | | | | | | | | | | |
| Anticoagulants | 57 (6.9) | 5,816 (5.8) | 0.19 | 7 (2.8) | 474 (3.1) | 0.9 | 34 (5.2) | 2,720 (4.9) | 0.74 | 0 | 21 (2.2) | 0.48 | 13 (1.8) | 639 (1.3) | 0.31 |  |
| Antiplatelets | 139 (17) | 18,937 (19) | 0.17 | 16 (6.3) | 1,238 (8.1) | 0.36 | 38 (5.8) | 3,713 (6.6) | 0.46 | 0 | 54 (5.6) | 0.1 | 44 (6.1) | 2,947 (6) | 0.97 |  |
| Antiarrhythmic agents | 448 (54) | 57,426 (57) | 0.14 | 110 (43) | 6,735 (44) | 0.88 | 225 (35) | 19,231 (34) | 0.95 | 13 (21) | 271 (28) | 0.28 | 149 (21) | 10,553 (22) | 0.63 |  |
| Lipid-modifying agents | 351 (42) | 48,798 (48) | <.001 | 85 (34) | 6,246 (41) | 0.02 | 168 (26) | 17,290 (31) | 0.006 | 11 (18) | 370 (38) | 0.002 | 284 (40) | 21,770 (44) | 0.009 |  |
| Antihypertensives | 490 (59) | 62,374 (62) | 0.16 | 120 (47) | 7,961 (52) | 0.15 | 291 (45) | 25,580 (46) | 0.64 | 16 (26) | 428 (44) | 0.006 | 218 (30) | 16,973 (35) | 0.02 |  |
| Rate-controlling agents | 475 (57) | 61,451 (61) | 0.05 | 120 (47) | 7,980 (52) | 0.14 | 271 (42) | 23,684 (42) | 0.76 | 14 (23) | 364 (38) | 0.02 | 196 (27) | 13,921 (28) | 0.52 |  |
| ACE inhibitors | 207 (25) | 29,586 (29) | 0.009 | 43 (17) | 3,156 (21) | 0.17 | 102 (16) | 9,837 (18) | 0.22 | 3 (4.8) | 164 (17) | 0.02 | 93 (13) | 7,095 (14) | 0.26 |  |
| ARBs | 146 (18) | 17,256 (17) | 0.69 | 42 (17) | 2,707 (18) | 0.7 | 78 (12) | 5,996 (11) | 0.33 | 5 (8.1) | 135 (14) | 0.26 | 60 (8.4) | 5,741 (12) | 0.006 |  |
| SGLT2 inhibitors | 24 (2.9) | 3,216 (3.2) | 0.72 | 6 (2.4) | 354 (2.3) | 1.0 | 12 (1.8) | 820 (1.5) | 0.53 | 0 | 33 (3.4) | 0.27 | 14 (1.9) | 1,085 (2.2) | 0.72 |  |
| Blood glucose regulation agents | 211 (26) | 29,132 (29) | 0.04 | 43 (17) | 3,597 (24) | 0.02 | 153 (24) | 13,084 (23) | 0.98 | 6 (9.7) | 293 (30) | <.001 | 116 (16) | 9,350 (19) | 0.05 |  |
| Immunosuppressants | 87 (11) | 8,701 (8.6) | 0.06 | 18 (7.1) | 1,408 (9.2) | 0.3 | 72 (11) | 4,660 (8.3) | 0.01 | 3 (4.8) | 130 (13) | 0.08 | 68 (9.5) | 4,018 (8.2) | 0.25 |  |
| ARNIs | 38 (4.6) | 4,606 (4.6) | 1.0 | 2 | 82 | 0.91 | 3 | 133 | 0.45 | 0 | 5 | 1.0 | 0 | 34 | 1.0 |  |
| Nitrates | 192 (23) | 23,269 (23) | 0.93 | 51 (20) | 3,090 (20) | 1.0 | 92 (14) | 7,461 (13) | 0.59 | 6 (9.7) | 150 (16) | 0.29 | 67 (9.3) | 6,249 (13) | 0.007 |  |
| Hydralazine | 43 (5.2) | 5,634 (5.6) | 0.7 | 13 (5.1) | 771 (5.1) | 1.0 | 23 (3.5) | 3,021 (5.4) | 0.04 | 0 | 29 (3) | 0.32 | 8 (1.1) | 568 (1.2) | 1.0 |  |
| MRAs | 100 (12) | 10,705 (11) | 0.18 | 19 (7.5) | 678 (4.4) | 0.03 | 42 (6.5) | 2,641 (4.7) | 0.05 | 2 (3.2) | 38 (3.9) | 1.0 | 23 (3.2) | 895 (1.8) | 0.01 |  |
| Steroids | 277 (33) | 28,554 (28) | <.001 | 92 (36) | 4,577 (30) | 0.03 | 260 (40) | 17,990 (32) | <.001 | 24 (39) | 377 (39) | 1.0 | 234 (33) | 13,351 (27) | 0.002 |  |
| Chemotherapeutics | 14 (1.7) | 673 | <.001 | 1 | 115 | 0.77 | 10 (1.5) | 427 | 0.04 | 1 (1.6) | 7 | 0.98 | 2 | 364 | 0.22 |  |
| Diuretics | 293 (35) | 34,623 (34) | 0.5 | 52 (21) | 2,966 (19) | 0.72 | 146 (22) | 11,466 (21) | 0.24 | 9 (15) | 131 (14) | 0.99 | 70 (9.7) | 3,870 (7.9) | 0.08 |  |
| Beta blockers | 426 (52) | 54,691 (54) | 0.15 | 101 (40) | 6,125 (40) | 0.99 | 188 (29) | 16,499 (30) | 0.77 | 9 (15) | 245 (25) | 0.08 | 129 (18) | 9,318 (19) | 0.5 |  |
| Warfarin | 46 (5.6) | 4,619 (4.6) | 0.2 | 5 (2) | 344 (2.3) | 0.93 | 16 (2.5) | 1,595 (2.9) | 0.63 | 0 | 11 (1.1) | 0.83 | 9 (1.3) | 460 | 0.5 |  |
| Direct OACs | 85 (10) | 11,844 (12) | 0.22 | 20 (7.9) | 1,027 (6.7) | 0.54 | 42 (6.5) | 4,170 (7.5) | 0.37 | 1 (1.6) | 53 (5.5) | 0.3 | 15 (2.1) | 1,105 (2.3) | 0.86 |  |
| Tafamidis | 0 | 5 | 1.0 | 0 | 0 | 1.0 | 0 | 0 | 1.0 | 1 (1.6) | 5 | 0.81 | 0 | 0 | 1.0 |  |
| **Cardiovascular laboratory results, median (IQR)** | | | | | | | | | | | | | | | | |
| BNP | 126 (51-518) | 204 (68-598) | 0.73 | 192 (88-229) | 88 (34-289) | 0.58 | 88 (35.2-253) | 121 (46-459) | 0.84 | 63.9 (63.9-63.9) | 57.6 (12.8-181) | n/a | 23.8 (17.9-29.6) | 53 (18.5-117) | 0.76 |  |
| NT-proBNP | 1,050 (104-4,450) | 976 (242-2,820) | 0.66 | 870 (870-870) | 335  (112-1,320) | n/a | 573 (285-1,520) | 464 (135-2,070) | 0.97 | n/p | 32 (23-4,750) | n/a | 42.5 (41.2-75.2) | 144 (51-395) | 0.76 |  |
| Cardiac troponin I | 0.051 (0-1.76) | 0.05 (0.02-0.28) | 0.88 | 0.0475 (0.03987-0.0563) | 0.05 (0.0145-0.138) | 0.77 | 0 (0-0.545) | 0.0333 (0.01-0.0795) | 0.91 | n/p | 0.03 (0.015-0.16) | n/a | n/p | 0 (0-0.04) | n/a |  |
| Cardiac troponin T | n/p | 0.105 (0.036-0.43) | n/a | n/p | 0.032 (0.0139-0.095) | n/a | 0 (0-0) | 0.0625 (0.0195-0.202) | n/a | 0 (0-0) | 0 (0-0) | 1.0 | n/p | 0.035 (0.0117-0.063) | n/a |  |
| LVEF | 37 (26-45) | 47 (35-57) | 0.08 | 65 (60-65) | 65 (58-70) | 0.78 | 57.5 (55-60) | 60 (55-65) | 0.68 | n/p | 67.5 (57.5-72.2) | n/a | 63.5 (58.8-67.5) | 60 (57-65) | 0.74 |  |
| HDL cholesterol | 44 (37-54.5) | 44 (35-54) | 0.46 | 46.5 (37.6-61.8) | 47 (38-58) | 0.77 | 47 (38-63.2) | 47 (37-59) | 0.49 | 52.5 (39.5-60.2) | 49.5 (41-61) | 0.75 | 50.5 (42.2-61.8) | 49 (40-61) | 0.11 |  |
| LDL cholesterol | 90.5 (63-113) | 89 (67-118) | 0.15 | 98 (75-120) | 97 (74-122) | 0.73 | 94 (72-120) | 95.3 (72-122) | 0.51 | 105 (91-128) | 95.5 (77-128) | 0.83 | 114 (84.8-160) | 114 (85-147) | 0.12 |  |
| Total cholesterol | 167 (134-192) | 165 (136-199) | 0.15 | 170 (142-198) | 175 (148-205) | 0.36 | 173 (147-203) | 176 (147-207) | 0.65 | 192 (167-202) | 176 (150-209) | 0.89 | 197 (164-244) | 195 (163-234) | 0.03 |  |
| Triglycerides | 117 (80.5-168) | 123 (87-180) | 0.1 | 99 (71-139) | 120 (86-171) | 0.02 | 119 (82-179) | 123 (86-182) | 0.47 | 91 (65.8-152) | 112 (82.8-160) | 0.2 | 112 (80-179) | 126 (88-182) | 0.56 |  |
| A1c | 6 (5.5-7.5) | 6.4 (5.7-7.9) | 0.23 | 5.8 (5.5-6.5) | 6 (5.6-7) | 0.22 | 5.8 (5.3-7.35) | 6 (5.5-7.5) | 0.17 | 5.7 (5.25-6.43) | 5.9 (5.5-6.9) | 0.26 | 5.6 (5.3-6.2) | 5.8 (5.5-6.7) | 0.005 |  |
| Fasting glucose | 91 (90-104) | 112 (94-149) | 0.1 | 94 (82.5-106) | 101 (90-123) | 0.51 | 93 (80.1-124) | 103 (89-137) | 0.19 | 101 (101-101) | 97 (88.2-122) | n/a | 86 (82-139) | 99 (89-117) | 0.34 |  |
| GFR | 75 (53-94) | 75 (54-94) | 0.52 | 73 (59.9-101) | 78.5 (60-97) | 0.84 | 85.5 (62-105) | 81.7 (56-104) | 0.08 | 81.2 (72.4-106) | 87 (67-104) | 0.92 | 90.8 (76.5-108) | 89.7 (74.5-104) | 0.33 |  |
| Hematocrit | 39.5 (34.2-43.9) | 40.5 (35.8-44.2) | 0.18 | 40.2 (35-44.5) | 40.8 (37-44) | 0.99 | 39.8 (34.5-42.7) | 39 (34-42.7) | 0.35 | 40.6 (35.6-44.4) | 40.7 (37.3-43.4) | 0.68 | 41.7 (37.6-44.5) | 41.4 (38.1-44.3) | 0.59 |  |
| Hemoglobin | 22 (14.4-23.5) | 22.3 (14.3-23.8) | 0.65 | 23 (20.8-24.2) | 22.4 (14.2-23.8) | 0.04 | 22.2 (14.2-23.6) | 22 (13.8-23.5) | 0.39 | 21.4 (14.3-23.3) | 22.4 (14.2-23.6) | 0.95 | 23.1 (18.6-24.1) | 23 (15.6-24) | 0.11 |  |
| INR | 38.3 (25.4-43.1) | 38.7 (21.3-43.4) | 0.29 | 40.3 (34.7-44.9) | 39.8 (33.7-43.6) | 0.27 | 38 (22.8-42.4) | 37.5 (19.3-42.1) | 0.02 | 39.8 (29.1-44.9) | 40.3 (35.8-43.3) | 0.96 | 41.7 (37.4-44.6) | 41.2 (37.9-44.4) | 0.51 |  |
| Platelet | 175 (112-260) | 178 (120-245) | 0.79 | 166 (111-239) | 181 (123-253) | 0.83 | 175 (121-250) | 176 (119-258) | 0.74 | 210 (136-270) | 191 (131-262) | 0.48 | 191 (132-266) | 185 (126-259) | 0.92 |  |
| Urine protein creatinine ratio | 332 (47.4-866) | 161 (6.29-1,080) | 0.7 | 85 (85-85) | 71.8 (0.39-335) | n/a | 524 (110-1,250) | 116 (1.58-1,090) | 0.94 | 3,320 (1,660-4,980) | 170 (77-480) | 0.38 | 102 (54.6-207) | 99.5 (3.14-252) | 0.04 |  |
| hsCRP | 1.63 (0.71-3.6) | 2.3 (0.9-6.32) | 0.54 | 2.1 (0.565-3.74) | 1.8 (0.7-5.19) | 0.51 | 4.65 (1.88-9.2) | 2.87 (1.02-6.39) | 0.67 | n/p | 1.3 (0.85-2.32) | n/a | 1.62 (0.775-3.5) | 1.61 (0.7-3.9) | 0.43 |  |
| ApoB | 84 (55.8-118) | 87 (69-106) | 0.99 | n/p | 76 (69.5-102) | n/a | 104 (82.6-106) | 89 (74.8-108) | 0.99 | n/p | 101 (101-101) | n/a | 95 (92-123) | 106 (89-134) | 0.67 |  |
| LP(a) | 2,200 (1,280-2,520) | 167 (22.9-2,900) | 0.74 | 46.8 (27.6-66.1) | 99.6 (22-2,510) | 0.33 | 5,170 (4,500-5,620) | 255 (55.5-3,490) | 0.009 | n/p | 10.5 (10.2-11.8) | n/a | 34.7 (4.62-1,640) | 152 (21.4-2,830) | 0.11 |  |

N = number of unique individuals; percentages indicate the proportion of records relative to the total of all unique records in the column. Abbreviations: DCM, dilated cardiomyopathy; HCM, hypertrophic cardiomyopathy; LQTS, long QT syndrome; FH, familial hypercholesterolemia; *P* = p value; HFpEF, heart failure with preserved ejection fraction; HFrEF, heart failure with reduced ejection fraction; TIA, transient ischemic attack; ACC, American College of Cardiology; AHA, American Heart Association; NYHA, New York Heart Association; EP, electrophysiology; ACE, angiotensin-converting enzyme; ARB, angiotensin receptor blocker; SGLT2, sodium-glucose cotransporter-2; ARNI, angiotensin receptor/neprilysin inhibitor; MRA, aldosterone receptor antagonists; OAC, oral anticoagulant; BNP, B-type natriuretic peptide; NT-proBNP, N-terminal-pro-BNP; LVEF, left ventricular ejection fraction; HDL, high-density lipoprotein; LDL, low-density lipoprotein; A1c, glycated hemoglobin; GFR, glomerular filtration rate; INR, international normalized ratio; hsCRP, high-sensitivity C-reactive protein; ApoB, apolipoprotein B; LP(a), lipoprotein(a); n/p, no laboratory results were present in the Veradigm Health Insights Ambulatory EHR Research Database linked with insurance claims data; n/a, not applicable, for laboratory results two-sample t-test could not be calculated as measurable only had one non-null value or had missing results (n/p) in tested or untested patients.

**Supplementary Figure 1. Geographic distribution of the study cohort across the United States.** No data was available for areas shown in gray.


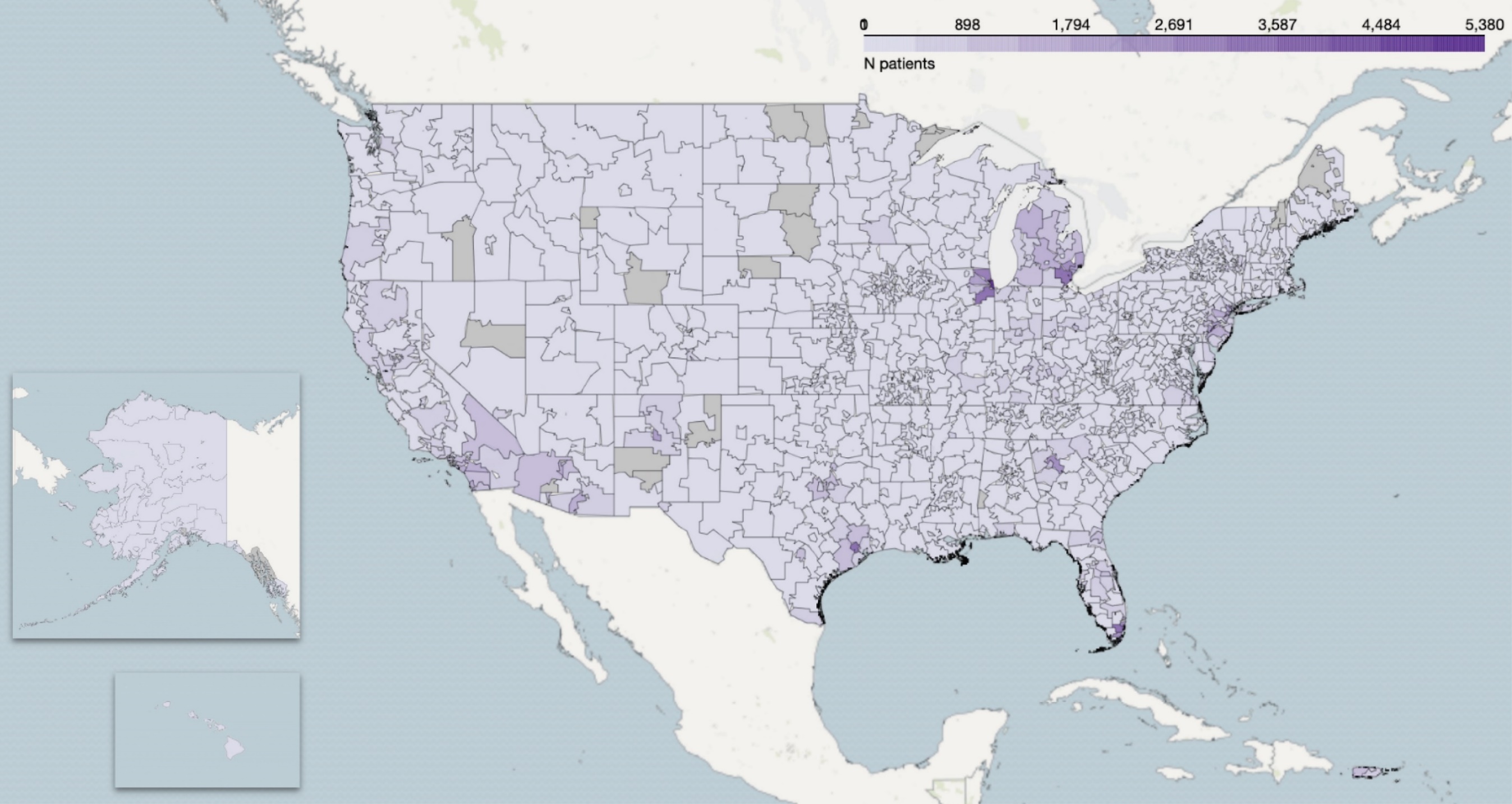


**Supplementary References**

1. Heidenreich PA, Bozkurt B, Aguilar D, Allen LA, Byun JJ, Colvin MM, et al. 2022 Aha/Acc/Hfsa Guideline for the Management of Heart Failure: A Report of the American College of Cardiology/American Heart Association Joint Committee on Clinical Practice Guidelines. *J Am Coll Cardiol* (2022) 79(17):e263-e421. Epub 20220401. doi: 10.1016/j.jacc.2021.12.012.

2. Al-Khatib SM, Stevenson WG, Ackerman MJ, Bryant WJ, Callans DJ, Curtis AB, et al. 2017 Aha/Acc/Hrs Guideline for Management of Patients with Ventricular Arrhythmias and the Prevention of Sudden Cardiac Death. *Circulation* (2018) 138(13):e272-e391. doi: 10.1161/cir.0000000000000549.

3. Bozkurt B, Colvin M, Cook J, Cooper LT, Deswal A, Fonarow GC, et al. Current Diagnostic and Treatment Strategies for Specific Dilated Cardiomyopathies: A Scientific Statement from the American Heart Association. *Circulation* (2016) 134(23):e579-e646. Epub 20161103. doi: 10.1161/CIR.0000000000000455.

4. Hershberger RE, Givertz MM, Ho CY, Judge DP, Kantor PF, McBride KL, et al. Genetic Evaluation of Cardiomyopathy-a Heart Failure Society of America Practice Guideline. *J Card Fail* (2018) 24(5):281-302. Epub 20180319. doi: 10.1016/j.cardfail.2018.03.004.

5. Ommen SR, Mital S, Burke MA, Day SM, Deswal A, Elliott P, et al. 2020 Aha/Acc Guideline for the Diagnosis and Treatment of Patients with Hypertrophic Cardiomyopathy: A Report of the American College of Cardiology/American Heart Association Joint Committee on Clinical Practice Guidelines. *J Am Coll Cardiol* (2020) 76(25):e159-e240. Epub 20201120. doi: 10.1016/j.jacc.2020.08.045.

6. Grundy SM, Stone NJ, Bailey AL, Beam C, Birtcher KK, Blumenthal RS, et al. 2018 Aha/Acc/Aacvpr/Aapa/Abc/Acpm/Ada/Ags/Apha/Aspc/Nla/Pcna Guideline on the Management of Blood Cholesterol: A Report of the American College of Cardiology/American Heart Association Task Force on Clinical Practice Guidelines. *Circulation* (2019) 139(25):e1082-e143. Epub 20181110. doi: 10.1161/CIR.0000000000000625.

7. Sturm AC, Knowles JW, Gidding SS, Ahmad ZS, Ahmed CD, Ballantyne CM, et al. Clinical Genetic Testing for Familial Hypercholesterolemia: Jacc Scientific Expert Panel. *J Am Coll Cardiol* (2018) 72(6):662-80. doi: 10.1016/j.jacc.2018.05.044.
